# Stable long-term germline transmission of GFP transgenic rat via *PiggyBac transposon* mediated gene transfer

**DOI:** 10.1186/s12917-024-04123-7

**Published:** 2024-06-26

**Authors:** Beom-Jin Jeon, Dong-Hyeok Kwon, Gyeong-min Gim, Hee-Kyoung Kim, Jeong-Hwa Lee, Goo Jang

**Affiliations:** 1 Laboratory of Theriogenology and Biotechnology, Department of Veterinary Clinical Science, College of Veterinary Medicine and the Research Institute of Veterinary Science, Seoul National University, 1 Gwanak-Ro, Gwanak-Gu, Seoul, 08826 Republic of Korea; 2 LARTBio Incorp, Gyeonggi-Do, Republic of Korea; 3 Comparative Medicine Disease Research Center, Seoul National University, Seoul, Republic of Korea; 4 Faculty of Veterinary Medicine, Universitas Airlangga, Surabaya, Indonesia; 5 K-BIO KIURI Center, Seoul National University, Seoul, Republic of Korea

**Keywords:** Germline Transmission, GFP-Rats, Resources, Transgene Silencing, Transposon

## Abstract

Transgene silencing provides a significant challenge in animal model production via gene engineering using viral vectors or transposons. Selecting an appropriate strategy, contingent upon the species is crucial to circumvent transgene silencing, necessitating long-term observation of in vivo gene expression. This study employed the PiggyBac transposon to create a GFP rat model to address transgene silencing in rats. Surprisingly, transgene silencing occurred while using the CAG promoter, contrary to conventional understanding, whereas the Ef1α promoter prevented silencing. GFP expression remained stable through over five generations, confirming efficacy of the Ef1α promoter for long-term protein expression in rats. Additionally, GFP expression was consistently maintained at the cellular level in various cellular sources produced from the GFP rats, thereby validating the in vitro GFP expression of GFP rats. Whole-genome sequencing identified a stable integration site in *Akap1* between exons 1 and 2, mitigating sequence-independent mechanism-mediated transgene silencing. This study established an efficient method for producing transgenic rat models using PiggyBac transposon. Our GFP rats represent the first model to exhibit prolonged expression of foreign genes over five generations, with implications for future research in gene-engineered rat models.

**Supplementary Information:**

The online version contains supplementary material available at 10.1186/s12917-024-04123-7.

## Introduction

Rats are larger than mice, allowing investigation periods, such as repeated medication delivery and blood collection. Due to these advantages, rats are commonly employed in research fields such as toxicology and neuroscience[1–8]. The advent of gene editing technologies such as ZFN, TALEN and CRISPR–Cas9 has recently reduced the limitations to making transgenic rats, resulting in the production of numerous transgenic rat models [9, 10]. Nonetheless, inserting a large-sized gene using these technologies remains problematic. Therefore, alternative ways of randomly integrating foreign genes into the host genome using viral vectors (such as lentivirus) or transposons (such as PiggyBac (PB) or Sleeping Beauty (SB)) are being developed. In particular, transposons, also known as jumping genes, have a greater cargo size (almost up to 200 kb) than viral vectors [11]. Moreover, no foreign proteins remain in the host cells, and their target site is ubiquitous across bacterial, plant, and animal genomes, making them suitable for producing a wide range of transgenic species [12–14].

However, transposons also create transgene silencing problems. Transgene silencing occurs for various reasons, but can be divided into sequence dependent and sequence independent mechanism [15]. The interaction between the transgenic circuit and host chromatin modifying enzymes causes sequence-dependent transgene silencing. However, sequence dependent transgene silencing can be avoided by selecting an adequate promoter for the chosen species. Sequence-independent mechanisms are caused by DNA methylation of the host genome integration locus, which is free in some genomic loci, known as safe harbor locus (e.g. ROSA26 locus in mice). However, whether transgene silencing occurs through a sequence-dependent or independent mechanism, developing a suitable method for each species to produce transgenic animals without transgene silencing is crucial.

Human elongation factor-1 alpha (Ef1α) and CMV-actin-globin (CAG) hybrid promoters are known as universal promoters used in mammals for transgene expression. However, Ef1α and CAG promoters are controversial for each species and cell type, especially in long-term transgene expression [16–18]. Although some in vitro studies have been conducted on the long-term expression of these two promoters, evidence from in vivo studies on long-term expression across generations remains limited. Thus, we established a GFP rat model using PiggyBac (PB) transposon to avoid transgene silencing through the sequence-dependent mechanism and evaluated the appropriate promoters for long-term transgene expression in a rat model. Moreover, whole genome sequencing (WGS) was performed to identify the gene loci required for long-term transgene expression without sequence-independent mechanism-mediated transgene silencing in rats.

## Materials and methods

### Animals

All animal care and procedures were approved by the Institutional Animal Care and Use Committee (No. SNU-201222–4-2) of Seoul National University Institute of Laboratory Animal Resources and perform under the guideline of Seoul National University. SD rats used in this study were purchased from Orient-bio (Seongnam, Republic of Korea) and maintained in 24 ± 2 °C, 50% humidity, and 12:12 h light–dark cycle (lights on from 07:00 to 19:00).

### Superovulation and embryo collection

Female rats were induced to superovulate by an intraperitoneal injection of 150 IU/kg PMSG (Daesung Microbiological labs, Republic of Korea) and 150 IU/kg hCG (Daesung Microbiological labs) with 48 h period, and then mated with males. Rats were anesthetized with intramuscular injection of 1.5 mg xylazine (Rompun®, Elanco Korea, Republic of Korea) and 0.35 mg alfaxalone (Alfaxan® multidose, Jurox, Australia) mixture and euthanized with cervical dislocation. One cell-stage embryos were collected from oviducts of females the day after mating in M2 medium (Sigma-Aldrich, USA), and cultured in mR1ECM medium (Cosmobio, Japan).

### Plasmid vector preparation

Piggybac empty vector and transposase vector were purchased from Addgene (http://www.addgene.org, Plasmids #20960) and Sanger Institute (Hinxton, UK) in previous studies[14, 19]. PB-CAG-GFP, PB-Ef1α-GFP, Ef1α-Transposase vectors were constructed using In-Fusion® HD Cloning Kit (TAKARA, Japan).

### Microinjection and embryo transfer

After removing the cumulus cells of embryos, transposon and transposase plasmids were microinjected into the cytoplasm by microinjector machine (Femtojet ®, Eppendorf, Germany) at 25 ng/µL concentration each. After injection, embryos were cultured in mR1ECM medium for 4 days. After 4 days, fifteen GFP expressing morula to early blastocyst stage embryos were transferred to the endometrium of pseudo-pregnant recipient females.

### CASA (Computer Assisted Sperm Analysis)

For sperm analysis, three GFP rats and three wild type rats of 8 weeks of age or older were used. After extracting the epididymis from an anesthetized rat by intramuscular injection of 1 mL of anesthetic (1.5 mg xylazine (Rompun®, Elanco Korea, Republic of Korea) and 0.35 mg alfaxalone (Alfaxan® multidose, Jurox, Australia) mixture), the epididymis was cut in M2 medium to obtain sperm. Three μl of appropriately diluted sperm were analyzed on leja 20 micron slide (Leja Products B.V., Noord-Holland, Netherlands), using IVOS II (Hamilton Thorne, Massachusettes, USA).

### Genotyping

Genomic DNAs for genotyping from all organs are extracted using DNeasy Blood&Tissue kit (Qiagen, Germany). PCR for GFP gene amplification are conducted using Mastercycler X50a (Eppendorf, Germany). Primers used in PCR are listed in Table 1.

**Table 1:** Primer list

Name	Direction	Sequence (5’—3’)
GFP—PCR	Forward	GCTCCCTTGGAGCCTACCTA
	Reverse	GTCCTCCTTGAAGTCGATGC
GFP—qRT-PCR	Forward	CTTTGTCCCAAATCTGGCGGA
	Reverse	GAAGTCGTGCTGCTTCATGTG
Actb—qRT-PCR	Forward	GCTACAGCTTCACCACCACA
	Reverse	TCTCCAGGGAGGAAGAGGAT
Akap1—qRT-PCR	Forward	GCTGGCTGAGAGGGTCAATT
	Reverse	TCAGGTGTGACAATCGCTCC

### Embryo freezing

Two-cell stage rat embryos were washed 3 times using M2 medium and gradually M2 medium-diluted BoviFreeze (minitube, Germany). Washed embryos were loaded in Ministraw (minitube) and frozen using Freeze control embryo freezers (CL8800i; minitube) by following the manufacturer’s protocol.

### Establishment of small intestinal and endometrial organoid

For the rat endometrial organoid culture, the middle part of jejunum and uterus from mature female rat was collected and minced into small pieces in 100 mm petri dish. After dissociation (Gentle Cell Dissociation Reagent, STEMCELL Technologies, Canada) reaction for 10 min on horizontal shaker at room temperature, mixture was washed with 1% BSA/PBS (Gibco, USA) and filtered through 70 µm cell strainer (SPL Life Sciences, Republic of Korea). Filtered mixture was centrifugated for 5 min at 1650 rpm. Pellet was suspended with 50% medium (IntestiCult™ Organoid Growth Medium (Human), STEMCELL Technologies) with Matrigel (CORNING, USA) to form dorm. The media was changed every 2–3 days and sub-cultured every 5–7 days.

### Rat embryonic fibroblast culture

Mated female rats were selected by checking plug, and anesthetized by intramuscular injection of 500µL of anesthetic (1.5 mg xylazine (Rompun®, Elanco Korea, Republic of Korea) and 0.35 mg alfaxalone (Alfaxan® multidose, Jurox, Australia) mixture). E14 embryos were isolated from endometrium and skin tissue of embryos were dissected and minced using surgical blades. Minced tissues were digested in 1 mL of 1U/mL Collagenase type I (Gibco)/HBSS media (Gibco) at 37℃ 5% CO_2_ atmosphere. Digested tissues were centrifuged in 500xg for 5 min and washed in 1 mL of PBS (Gibco), and then, cultured in 10% FBS (Gibco) DMEM (Cytiva, USA) at 37℃ 5% CO_2_ atmosphere.

### Library construction and sequencing

Genomic DNA was extracted from tail of GFP F6 rats using QIAamp DNA Mini Kit (Qiagen). The quality and quantity of purified DNA were assessed by fluorometry (Qubit, Invitrogen) and gel electrophoresis. Briefly, 100 ng of genomic DNA from each samples were fragmented by acoustic shearing on a Q800R2 instrument (Qsonica, USA). Fragments of 350 bp were ligated to Illumina’s adapters and PCR-amplified (TruSeq® Nano DNA Library Prep Kit, Illumina, USA). 500-600 bp is appropriate size for final library. Libraries were quantified using the TapeStation 4200 instrument (Agilent Technologies, USA) and KAPA Library Quantification Kit (Kapa Biosystems, USA). The resulting purified libraries were applied to an Illumina flow cell for cluster generation and sequenced using 150 bp paired-end reads on an Illumina NovaSeq 6000 (Illumina) sequencer by following the manufacturer’s protocols.

### Sequencing data quality control

Over about six hundred million pass-filter reads were generated per each sample. Quality control analysis of the sequencing reads was conducted using the FastQC software (version 0.10.1) [20]. Trimming and read filtering was conducted using sickle software (version 1.3.3) [21]. Briefly, reads containing N more than 10% of the sequence, more than 40% of bases below Q20 or average quality below Q20 were filtered and discarded.

### Read mapping and variant analysis

Filtered reads were mapped to the reference rattus norvegicus genome sequence (mRatBN7.2) with minimum seed value 45 using BWA software (version 0.7.17) [22]. Duplicated reads are removed to avoid overweighting of some genomic locus caused by inhomogenoerous PCR amplification using GATK software (version. 4.0.2.1) [23]. Duplication removed sequence were used for variant analysis using GATK software, with option as follow, stand_call_conf – 30.0, stand_emit_conf – 10.0, dcov – 1,000. To predict the functional effects of the variants detected from GATK, SnpEff software (version 4.1) [24] and mRatBN7.2/Rattus norvegicus Ensembl annotation were used.

### Integration site analysis

Transgene were aligned and mapped to aligned data from BWA. Duplicated sequencing reads removal was perform on aligned bam file using picard. After extracting soft-clipped reads, alignment is performed by BLAST with rattus norvegicus genome as a reference for the soft-clipped reads and this site was expected to be an integration site. To validate the integration site, we placed span reference genome on both sides of transgene and made hybrid sequence.

### qRT-PCR

Total RNAs were extracted from GFP rat tail and liver tissue using RNeasy mini kit(Qiagen). Five ug of total RNA was used for synthesizing cDNA using RNA to cDNA EcoDry™ Premix Kit (Clontech, USA). qRT-PCR was conducted in MicroAmp™ Optical 96-Well Reaction Plate (Applied Biosystems, USA) using TB Green® Premix Ex Taq™ (TAKARA). qRT-PCR was conducted using Quantstudio 3 Real-Time PCR instrument (Applied Biosystems). Target gene relative expression was normalized to Actb expression using the comparative CT (2^− ΔΔCt^) method. Primers used in qRT-PCR are listed in Table 1.

### Statistical analysis

Data from CASA were statistically analyzed using student’s t-test, which were performed using GraphPad Prism version 8.0.1 for Windows (GraphPad Software, USA, www.graphpad.com). When *p* value was lower than 0.05, results were considered as statistically significant.

## Results

### Production of transgenic rats with PiggyBac transposon and Ef1α promoter

**Fig. 1:**
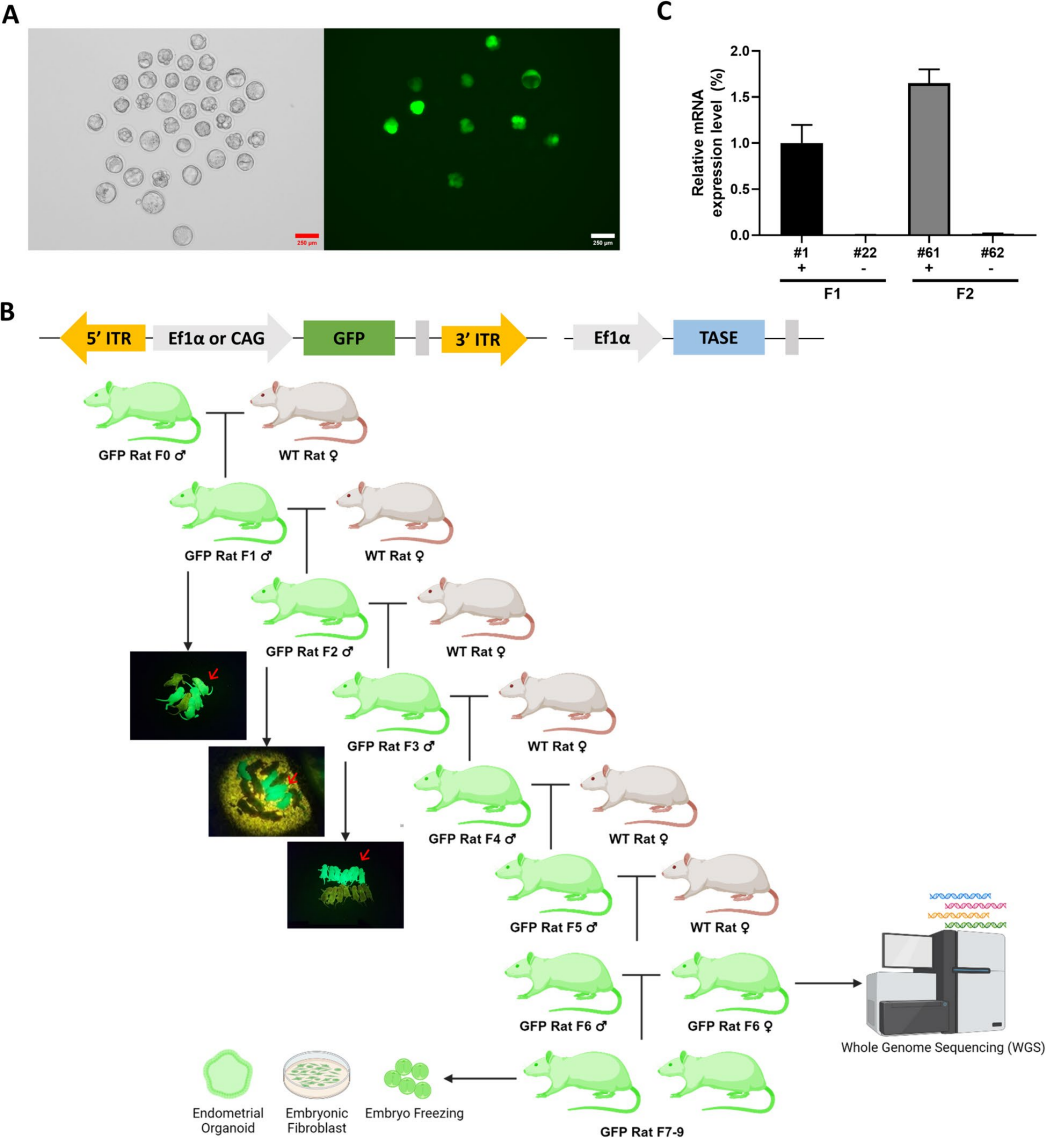
Generation and establishment of PB-Ef1α-GFP rat line. **A** Representative image of PB-Ef1α-GFP rat embryos. Scale bars = 250 µm. **B** Schematic figure of GFP rat production and analyzation. Strongly expressing rats (red arrow) are selected for next generation production. F6 male and female rats are used for whole genome sequencing (WGS) and following generations are used for establishing biological resources. ITR: Inverted terminal repeat sequences. TASE: Transposase. **C** Representative GFP qRT-PCR result of GFP rats. + , visually GFP positive; -, visually GFP negative. Full graph is on supplementary figure

First, we examined the efficiency of CAG and Ef1α promoters by microinjecting a PB transposon vector containing each promoter (PB-CAG-GFP and PB- Ef1α-GFP) and transposase vector (Ef1α-TASE) into one-cell stage rat embryos. When rats were produced by injecting the PB-CAG-GFP vector, GFP was not expressed in blastocysts (Supplementary Fig. 1A) and offspring (data not shown). To determine whether PB transposon had been integrated into the genome, gDNA was extracted from the tails of the pups, and the GFP gene was confirmed by PCR analysis. Surprisingly, the GFP gene was integrated (Supplementary Fig. 1B). However, because GFP had not been visually validated in the pups, fibroblasts were isolated from the PCR-positive pups, although GFP expression was again not detected (Supplementary Fig. 1C). To determine whether the problem was with the PB-CAG-GFP vector, HeLa cells were transfected with the PB-CAG-GFP vector and Ef1α-Transposase vector. Subsequently, GFP was shown to be expressed normally (Supplementary Fig. 1D). Additionally, the same vector expressed normally in our previous studies for long-term [25, 26]. These results imply that the PB-CAG-GFP vector has a transgene silencing problem in rats. The PB-Ef1α-GFP vector was also evaluated, and unlike the PB-CAG-GFP vector, GFP was found to be expressed in blastocysts and offspring (Fig. 1A, C). Since gene integration with the PiggyBac transposon occurs at multiple locations in the host genome, and we assumed that at least one of these loci was required for long-term GFP expression, GFP males were selected via qRT-PCR and mated with wild-type females for up to six generations to reduce the PB-Ef1α-GFP integration locus (Fig. 1B, C, Supplementary Fig. 1E).

### Validation of long-term GFP expression and reproductive ability

**Fig. 2:**
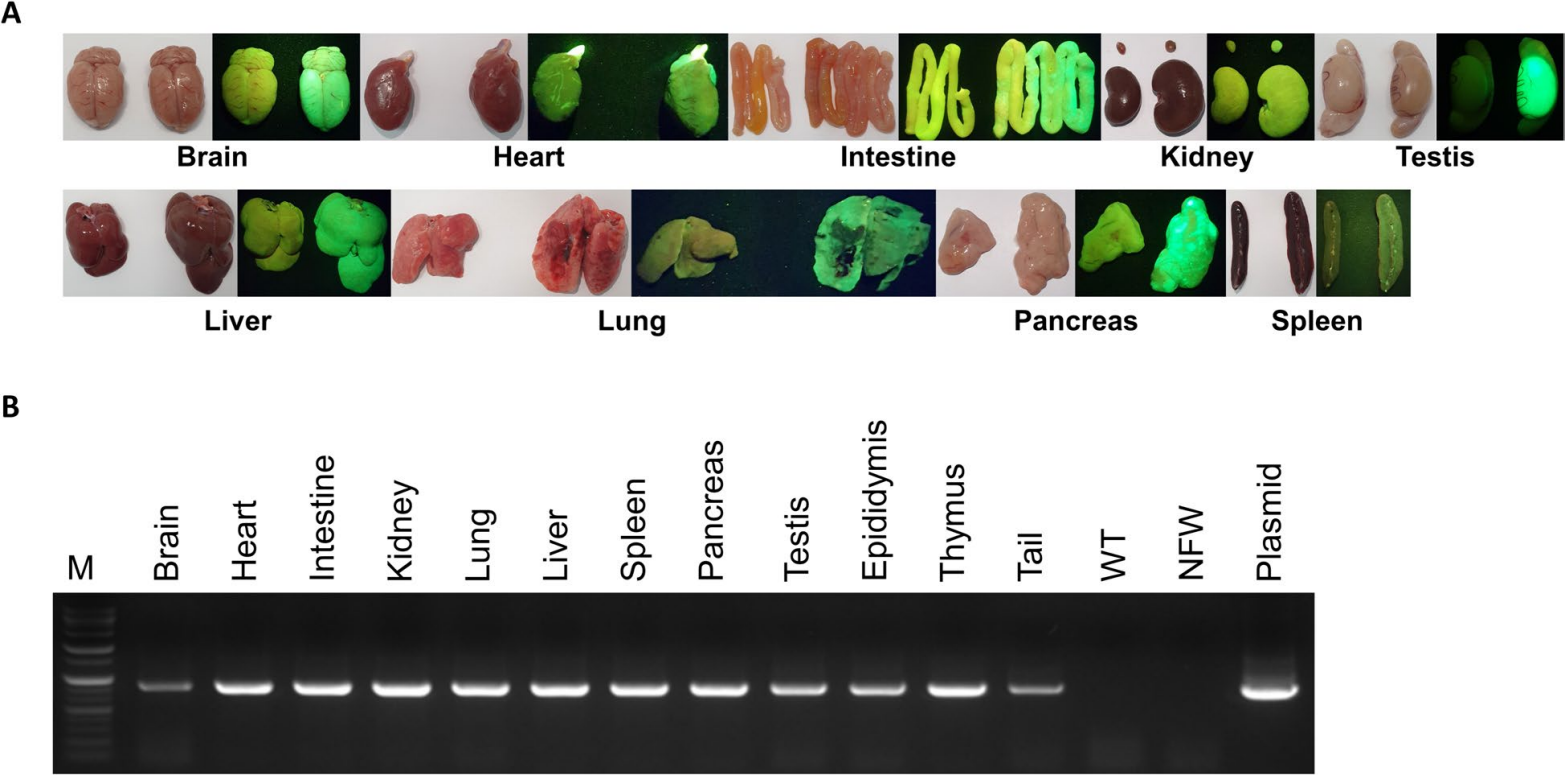
Validation of long-term GFP expression. **A** Images of wild-type(WT) and GFP rat organs under bright light or fluorescence lamp. **B** PCR result of GFP gene using GFP organs. M, marker; WT, wild-type rat tail; NFW, nuclease free water (negative control); Plasmid, PB-Ef1α-GFP vector (positive control)

To validate the long-term expression of PB- Ef1α-GFP, 83-week-aged GFP F_0_ and 26-week-old wild-type rat organs were removed and analyzed using fluorescent lamps and PCR. The GFP rat organs were larger overall, which is assumed to be related to the age differences between animals. Even though one-cell stage zygote microinjection is prone to mosaicism [27], all organs normally expressed GFP (Fig. 2A). Additionally, the presence of the GFP gene was validated using PCR, with all organs confirmed to possess the GFP gene in their genomes (Fig. 2B). Although GFP is usually considered to be stable in mammalian cells, previous research studies found that the EGFP (or just GFP) employed in our study induces oxidative stress and cytotoxicity [28, 29]. Thus, we conducted computer-assisted sperm analysis (CASA) and produced offspring to ensure stable germline transmission in the produced GFP rats. When sperms were analyzed using CASA, no significant difference was identified between the GFP rat sperm and wild-type sperm (Table 2); moreover the GFP phenotype was transmitted to the next generation without the concern of transgene silencing occurring during germline transmission.

**Table 2:** Computer Assisted Sperm Analysis (CASA) results of GFP male rat

Variable	GFP (*n* = 3)	Wild type (*n* = 3)	*P*-value
Motile cells (%)	60.15 ± 10.41	61.63 ± 4.87	0.4471
Progressive cells (%)	26.08 ± 8.14	22.78 ± 2.56	0.3590
VAP (µm/s)	232.85 ± 19.85	235.68 ± 5.79	0.4502
VCL (µm/s)	443.24 ± 35.99	437.49 ± 9.78	0.4492
VSL (µm/s)	181.32 ± 28.36	171.24 ± 5.52	0.3692
ALH (µm)	32.43 ± 3.03	29.95 ± 0.98	0.2235
BCF (Hz)	20.26 ± 2.84	20.92 ± 1.63	0.4032
LIN (VSL/VCL)	39.06 ± 3.46	38.62 ± 1.37	0.4433
STR (VSL/VAP)	76.92 ± 7.41	71.28 ± 1.84	0.2572

### Establishment of GFP cellular resources

**Fig. 3:**
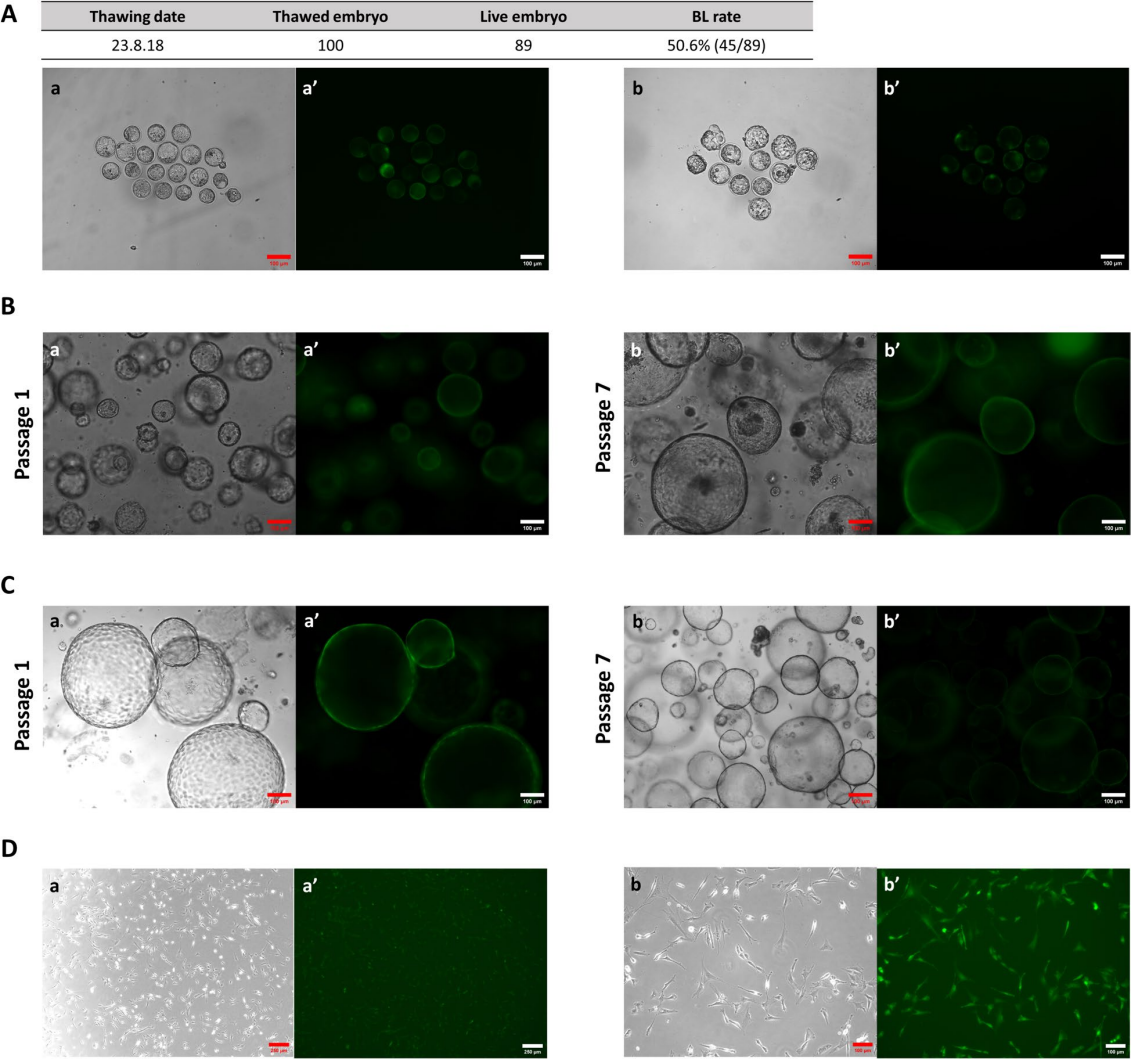
Establishment of cellular entities from GFP rat line. **A** Thawing information of frozen GFP rat embryo. (a-a’) Representative images of non-frozen GFP blastocyst. (b-b’) Representative images of frozen-thawed GFP blastocyst. Scale bars = 100 µm. **B** Representative images of endometrial organoid from GFP rat adult stem cell. (a-a’) Organoids of passage 1. (b-b’) Organoids of passage 7. Scale bars = 100 µm. **C** Representative images of small intestinal organoid from GFP rat adult stem cell. (a-a’) Organoids of passage 1. (b-b’) Organoids of passage 7. Scale bars = 100 µm. **D** Representative images of GFP rat embryonic fibroblast. (a-a’) Images of 40 × magnification. Scale bars = 250 µm. (b-b’) Images of 100 × magnification. Scale bars = 100 µm

To verify in vitro GFP expression in the GFP rat and preserve the diverse types of GFP-expressing cells, we established and cryopreserved several cellular entities. First, fertilized embryos were retrieved and two-cell stage embryos were frozen to reduce animal farming and preserve the GFP rat line for future research. Furthermore, cryopreserved embryos were thawed to confirm GFP expression and survival rate in the frozen embryos. When blastocyst formation rate and GFP expression were measured, most of the thawed embryos survived (89%; 89/100), with approximately 50% developing into blastocysts (45/89), which stably expressed GFP (Fig. 3A). In addition, to confirm GFP expression in several cell types, organoids were cultivated using adult stem cells from the endometrium and small intestine, and embryonic fibroblasts were cultured using fetal epithelium. GFP persistently expressed in a various cells from different organs (epithelial cells, epithelial stem cells, stromal cells, intestinal stem cells, etc.; Fig. 3B-D). From these cells, we confirmed that GFP rats generated using PB-Ef1α-GFP stably express GFP both in vivo and in vitro.

### Whole genome analysis of GFP rats

**Fig. 4:**
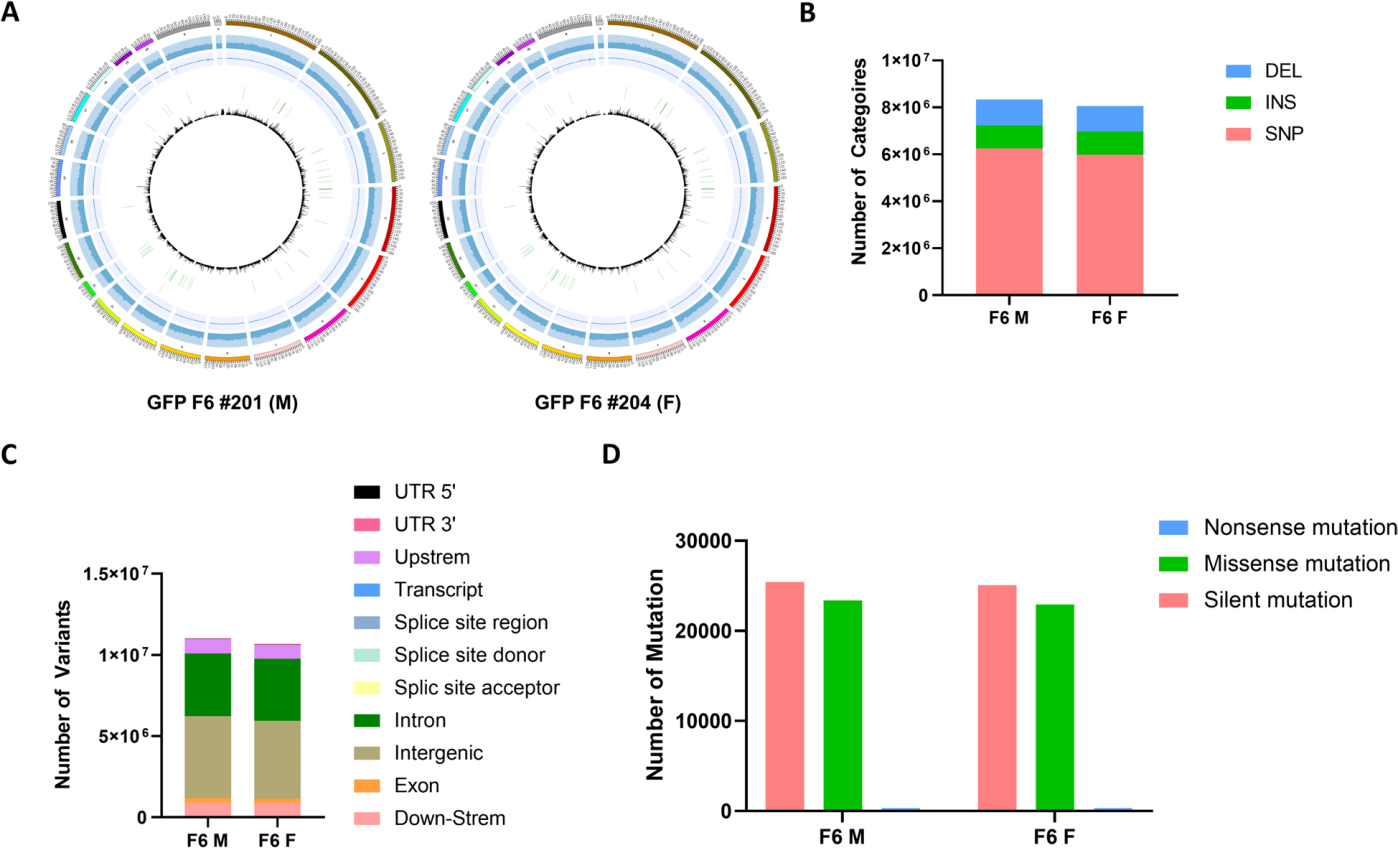
Whole genome analysis of GFP rat line. **A** Circos plot of GFP rat whole genome sequencing (WGS) results. Chromosome, GC rate histogram, GC skew line plot, integration site, histogram of the number of rRNA and tRNA, and variants number histogram (bin size = 0.1 Mb) from outside to inside. **B** Graph of the number of SNP and INDEL. DEL: Deletion, INS: Insert, SNP: Single nucleotide polymorphism. **C** Graph of genomic variatns. **D** Graph of mutation effects by functional classes

We assumed this stable GFP expression were due to integrating the PB-Ef1α-GFP transgene into a specific genome site. To confirm our hypothesis, GFP males were constantly bred with wild-type female, and whole genome sequencing(WGS) was performed using F_6_ generation GFP rats in which about 50% of the litter expressed GFP (Fig. 1B). The analysis results showed that GFP F_6_ male and female rats had similar SNP and INDEL patterns, as expected given that they were backcrossed with wild-type females for six generations (Fig. 4B, Table 3). Likewise, they had similar patterns of genomic variants and mutation effects according to functional class (Fig. 4C, D). Overall, circos plots showed genomic similarity in GC, RNA and variant contents at the chromosome level (Fig. 4A). These data show our GFP rats have genomic stability through repeated generations. Importantly, they had the same integration locus in the *Akap1* intron between exon 1 and 2 (Fig. 1, Table 4); thus, we assume this locus is crucial for long-term transgene expression. Because both the transgene (PB-Ef1α-GFP) and transposase (Ef1α-Transposase) circuits were injected, there were concerns that transgene jumping would occur following the random integration of the Ef1α-Transposase vector. Fortunately, the WGS results indicated that the Transposase vector was not integrated (data not shown), and transgene integration and expression in *Akap1* were confirmed until the F_10_ generation (Supplementary Fig. 2A). To confirm whether transgene integration interferes with gene expression at the integration locus, qRT-PCR was conducted, and *Akap1* expression was shown to increase by 15-fold (Supplementary Fig. 2B).

**Table 3:** Integration site analysis

I.D	SNP	INS	DEL
GFP F6 #201	6249018	976335	1105999
GFP F6 #204	5988246	973198	1094523

**Table 4:** Integration site analysis

I.D	**Chromosome**	**Insert Site**	**Orientation**	**Overlapping gene**	**Location**	**5’gene**	**3’gene**
GFP F6 #201	NC_051345.1	73,640,204–73,640,205	Reverse	Akap1	E1-2 intron	Akap1	Akap1
GFP F6 #204	NC_051345.1	73,640,204–73,640,205	Reverse	Akap1	E1-2 intron	Akap1	Akap1

## Discussion

Transgene silencing is one of the major issues in using transposons to produce transgenic animals[30]. In a previous study [31], it was shown that the CAG promoter–GFP vector was well-expressed in rats; however, in our study, when CAG-GFP was used with PB, GFP expression was not observed although GFP gene integration into the host genome and gene silencing issues were occurred. In the previous study, vector injection into the pronucleus was performed, whereas in our study, cytoplasmic injection with PB was employed, which is considered to have resulted in different outcomes. Thus, this phenomenon is assumed to occur when the PB and CAG promoters are combined in rat embryos rather than a problem with the CAG promoter itself. Unlike the CAG promoter, when GFP was used with the Ef1α promoter, GFP expression was intensive and lasted long-term. Despite concerns about germline transmission transgene silencing and mosaicism from using the one-cell stage microinjection and transposon vector, our GFP rat showed long-term and multiple-generation GFP expressions throughout the body. Therefore, from these results, we found that the Ef1α promoter is the more effective strategy to avoid sequence-dependent transgene silencing than the CAG promoter when using PB in rat embryos.

Since we anticipated that specific genomic locus was evitable for these stable PB-Ef1α-GFP expressions, avoiding sequence-independent transgene silencing, we crossed GFP male rats with WT females and selected strongly expressing offspring for the next generation until the GFP positive rat ratio became 50%, and conducted whole-genome analysis using WGS on F_6_ rats. As a result, both pups showed consistent gene expression when inserting the PB-Ef1α-GFP into the intergenic region of *Akap1*. Thereafter, when the *Akap1* expression was validated following transgene integration, it was found to be uninterrupted, meaning the expression of *Akap1* was increased, maybe due to an enhancer effect by the Ef1α promoter. The fact that the inserted gene is expressed throughout multiple generations suggest that the integration site might be a safe harbor locus candidate. However, to verify it as a safe harbor locus, inserting of a foreign gene into the locus must not adversely affect the individual’s health [32, 33]. According to some studies, an increase in *Akap1* has been detected in cancer patients [34, 35], with studies demonstrating that *Akap1,* which is a target of proto-oncogene, *Myc*, stimulates the mTOR pathway[33], resulting in the development of cancer. However, limited research exists in animal models investigating how cancer occurs when the *Akap1* expression is enhanced. Although our GFP rats grew normally until 83 weeks, we did not perform specific tissue histology or cancer development investigations. Therefore, to utilize the intergenic region of *Akap1* as a safe harbor locus in future studies, more researches on the carcinogenic effect of *Akap1* overexpression is necessary.

To summarize, by creating transgenic rats using the PiggyBac transposon, we have identified an effective way to achieve long-term and multiple-generation protein expressions without transgene silencing. Specifically, the Ef1α promoter was shown to be more effective than CAG, and it was verified for the first time that this long-term expression could be obtained in vivo for over 10 generations. The diverse cellular sources derived from our PB-Ef1α-GFP rats are valuable for future investigations. While WGS verified the stability of transgene expression in *Akap1*, its impact on the organism remains uncertain and more research is required.

## Conclusion

We addressed reproductive health issues and transgene silencing considerations by establishing the PB-Ef1α-GFP rat line using the PB transposon. This research showed long-term and multiple-generation (over five generations) in vivo transgene expression for the first time in transgenic rats. It is proven that different biological resources (including organoids and embryonic fibroblasts) maintained high levels of GFP expression, demonstrating that in vitro silencing did not occur. Using WGS, we definitively validated the stable genomic locus of Akap1. The discoveries in these transgenic rats can be used as a basis for further investigation into the development of genetically modified rats.

### Supplementary Information

Supplementary Material 1.

Supplementary Material 2.

## Data Availability

The datasets generated and/or analysed during the current study are available in the sequence read archive (SRA) of national center for biotechnology information (NCBI), SAMN38935809, SAMN38935810. The other materials are available from the corresponding author on reasonable request.
