# Supplementary material for: Stable long-term germline transmission of GFP transgenic rat via PiggyBac transposon mediated gene transfer
Source: BMC Vet Res. 2024 Jun 26;20:275. doi: 10.1186/s12917-024-04123-7 (PMC11201299; doi:10.1186/s12917-024-04123-7)
Supplement: Supplementary file 1 — Supplementary Material 1. [file 12917_2024_4123_MOESM1_ESM.docx]

**Supplementary information**

99

100

101

102

103

104

105

106

107

108

109

110

111

112

113

114

WT

NFW

PC


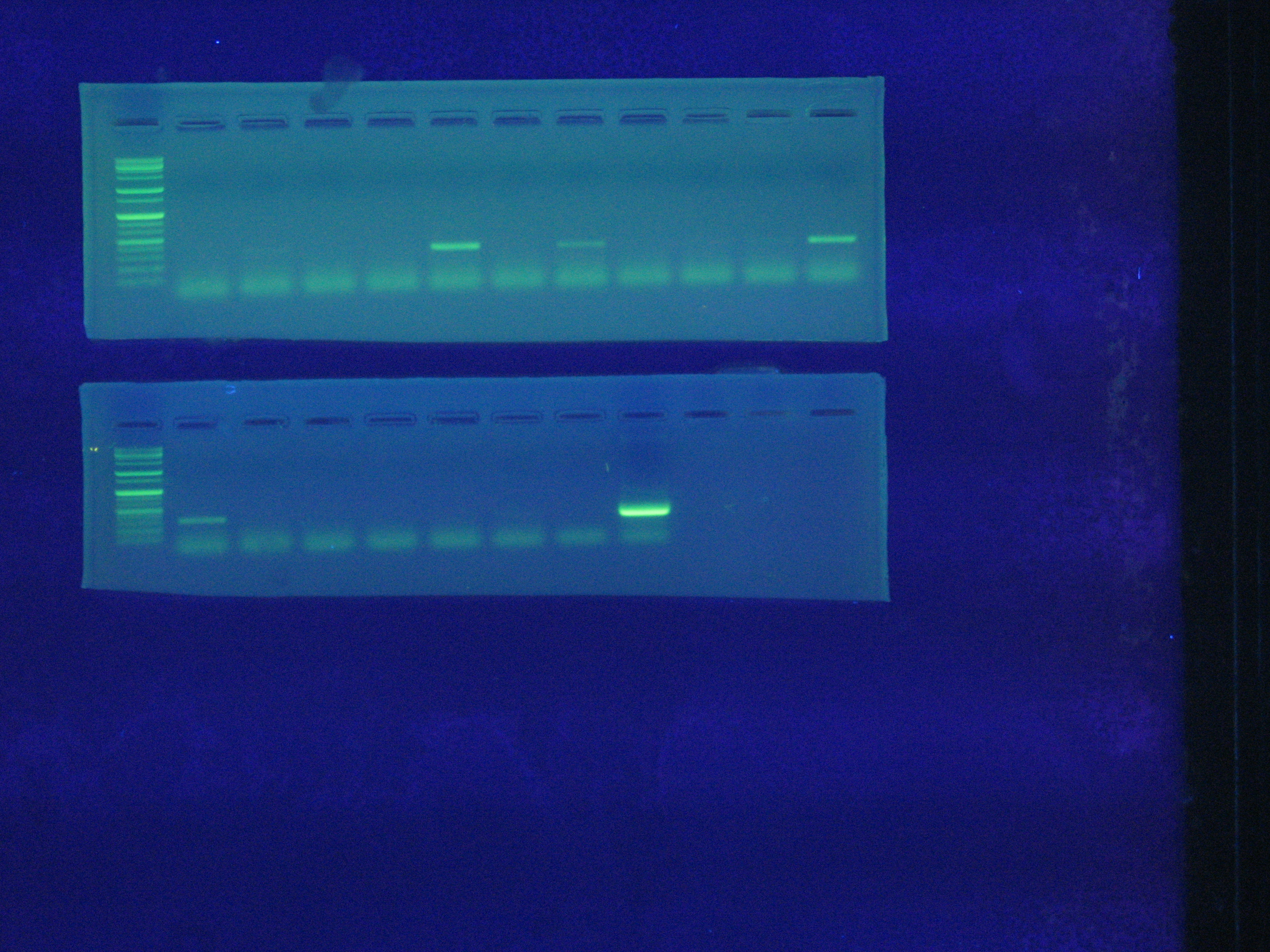


Supplementary Information 1. Full-length image of Supplementary figure 1B. Top, left image of Supplementary Fig 1B. Bottom, right image of Supplementary Fig. 1B. WT: wild-type rat tail, NFW: nuclease free water, negative control, PC: Positive control

**
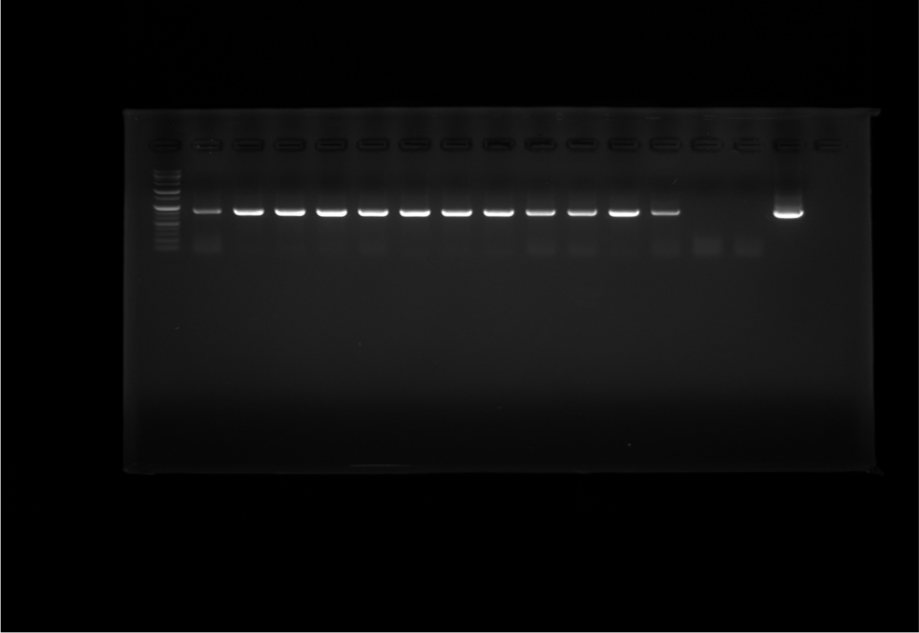
**

Brain

Heart

Intestine

Kidney

Liver

Lung

Pancreas

Spleen

Testis

Tail

Thymus

WT

NFW

Plasmid

Epididymis

M

Supplementary Information 2. Full-length image of Figure 2B. M: marker, WT: wild-type rat tail, NFW: nuclease free water, negative control, Plasmid: positive control

**
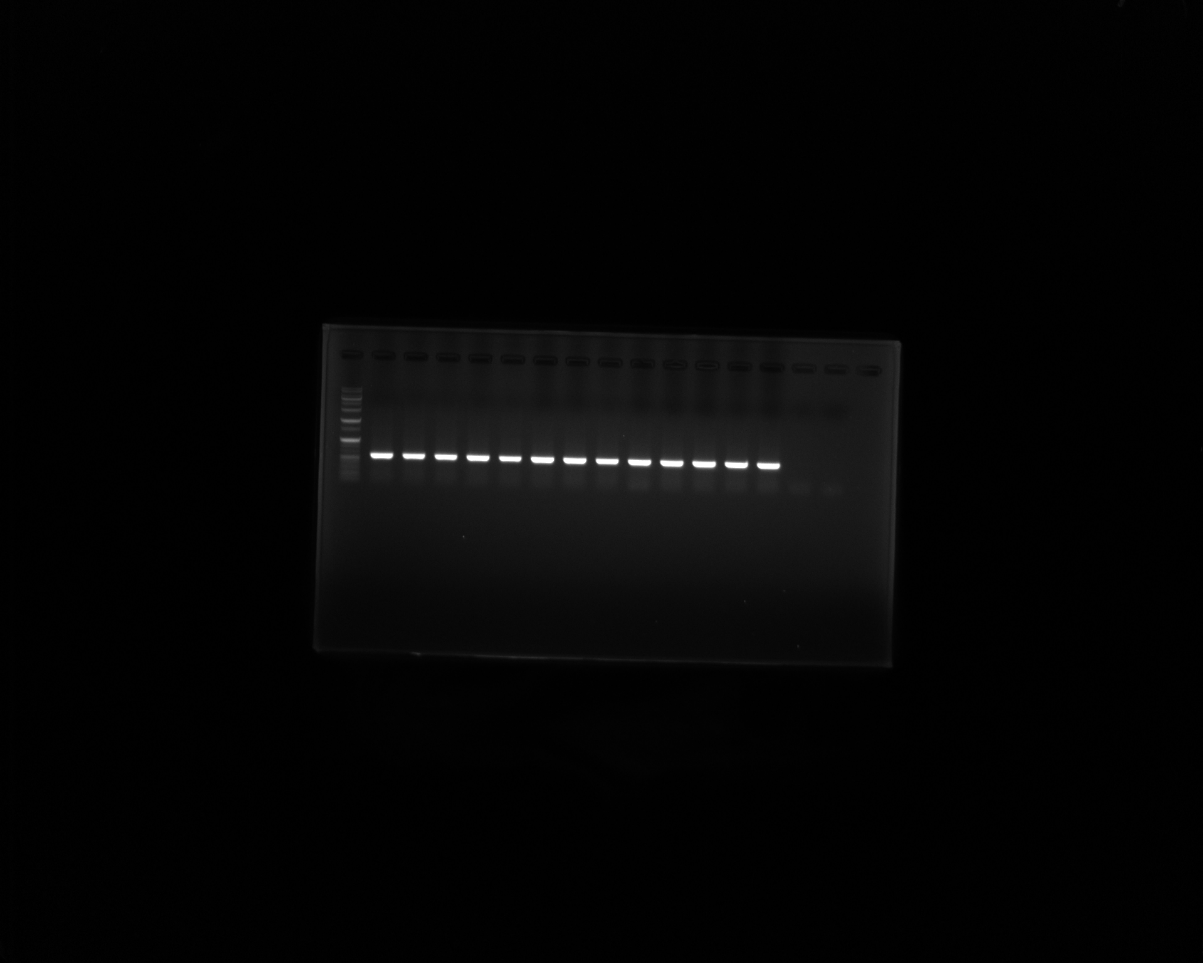
**

**M**

**349**

**350**

**351**

**352**

**353**

**354**

**355**

**356**

**357**

**358**

**359**

**360**

**361**

**WT**

**NFW**

**M**

**349**

**350**

**351**

**352**

**353**

**354**

**355**

**356**

**357**

**358**

**359**

**360**

**361**

**WT**

**NFW**


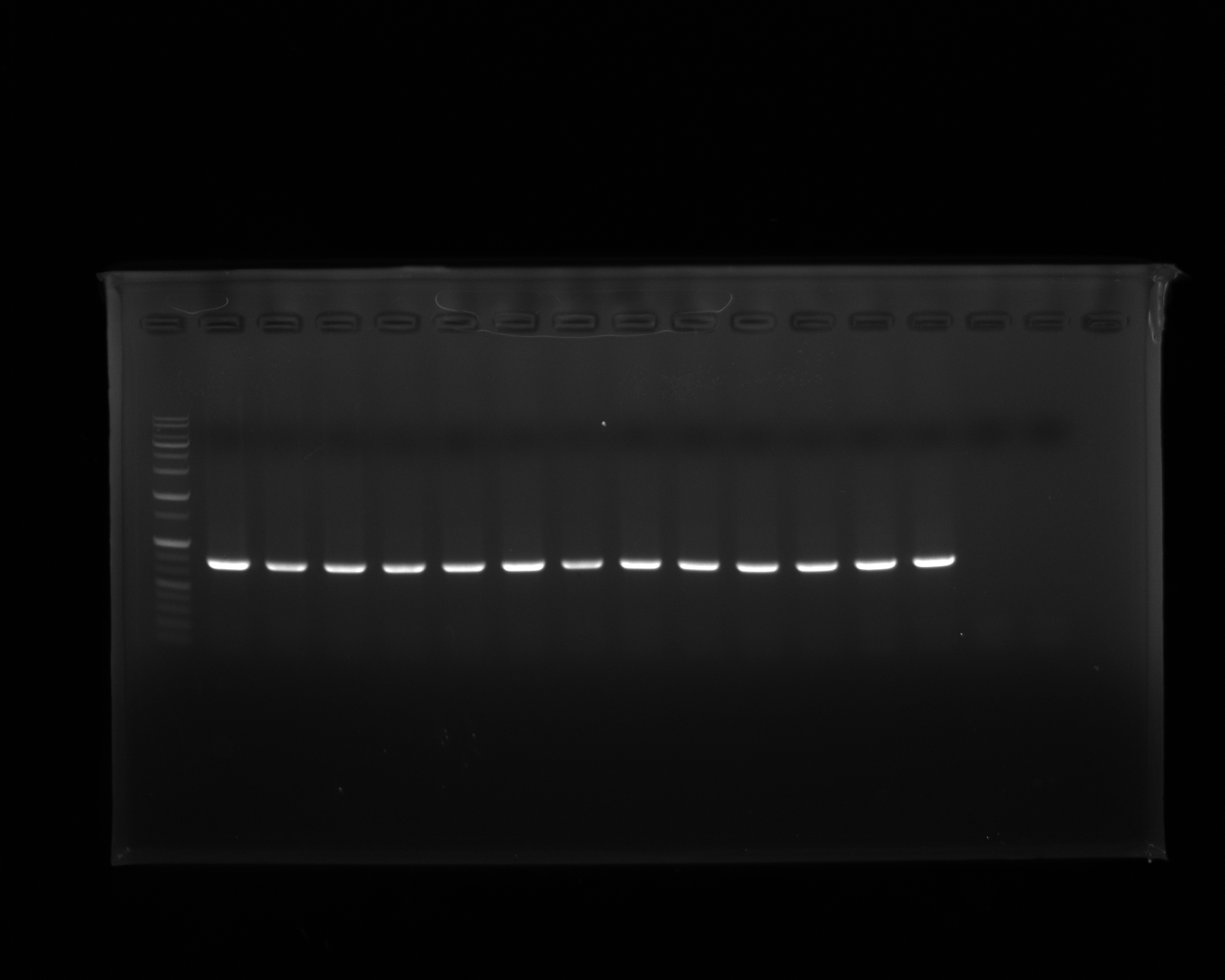


**M**

**349**

**350**

**351**

**352**

**353**

**354**

**355**

**356**

**357**

**358**

**359**

**360**

**361**

**WT**

**NFW**


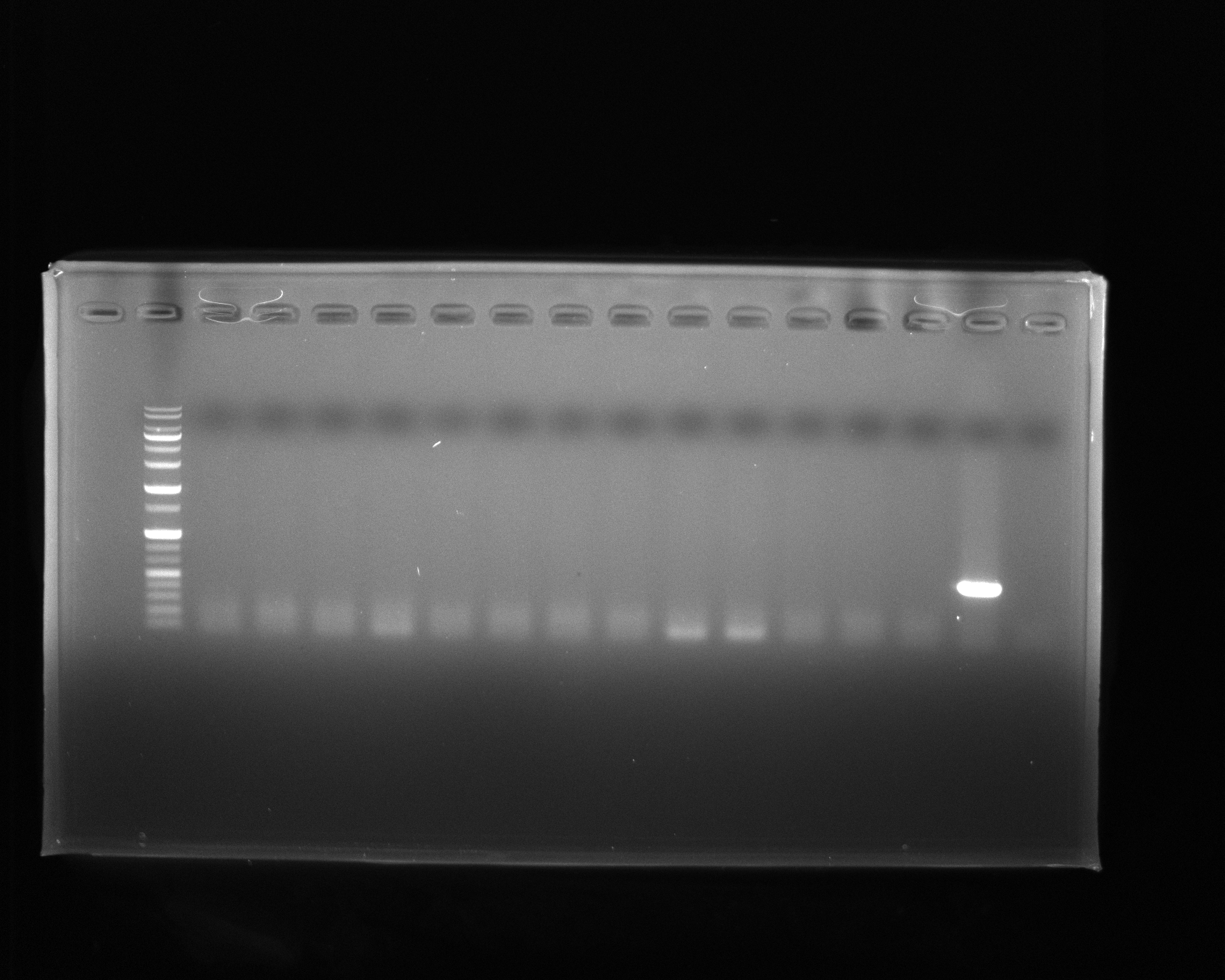


Supplementary Information 3. Full-length image of Supplementary figure 2A. Top: first gel image of Supplementary Fig. 2A, Middle: second gel image of Supplementary Fig. 2A, Bottom: third gel image of Supplementary Fig. 2A. M: marker, WT: wild-type rat tail, NFW: nuclease free water, negative control, Plasmid: positive control
