# Supplementary material for: Stable long-term germline transmission of GFP transgenic rat via PiggyBac transposon mediated gene transfer
Source: BMC Vet Res. 2024 Jun 26;20:275. doi: 10.1186/s12917-024-04123-7 (PMC11201299; doi:10.1186/s12917-024-04123-7)
Supplement: Supplementary file 2 — Supplementary Material 2. [file 12917_2024_4123_MOESM2_ESM.docx]

**Supplementary figures**

Supplementary figure 1


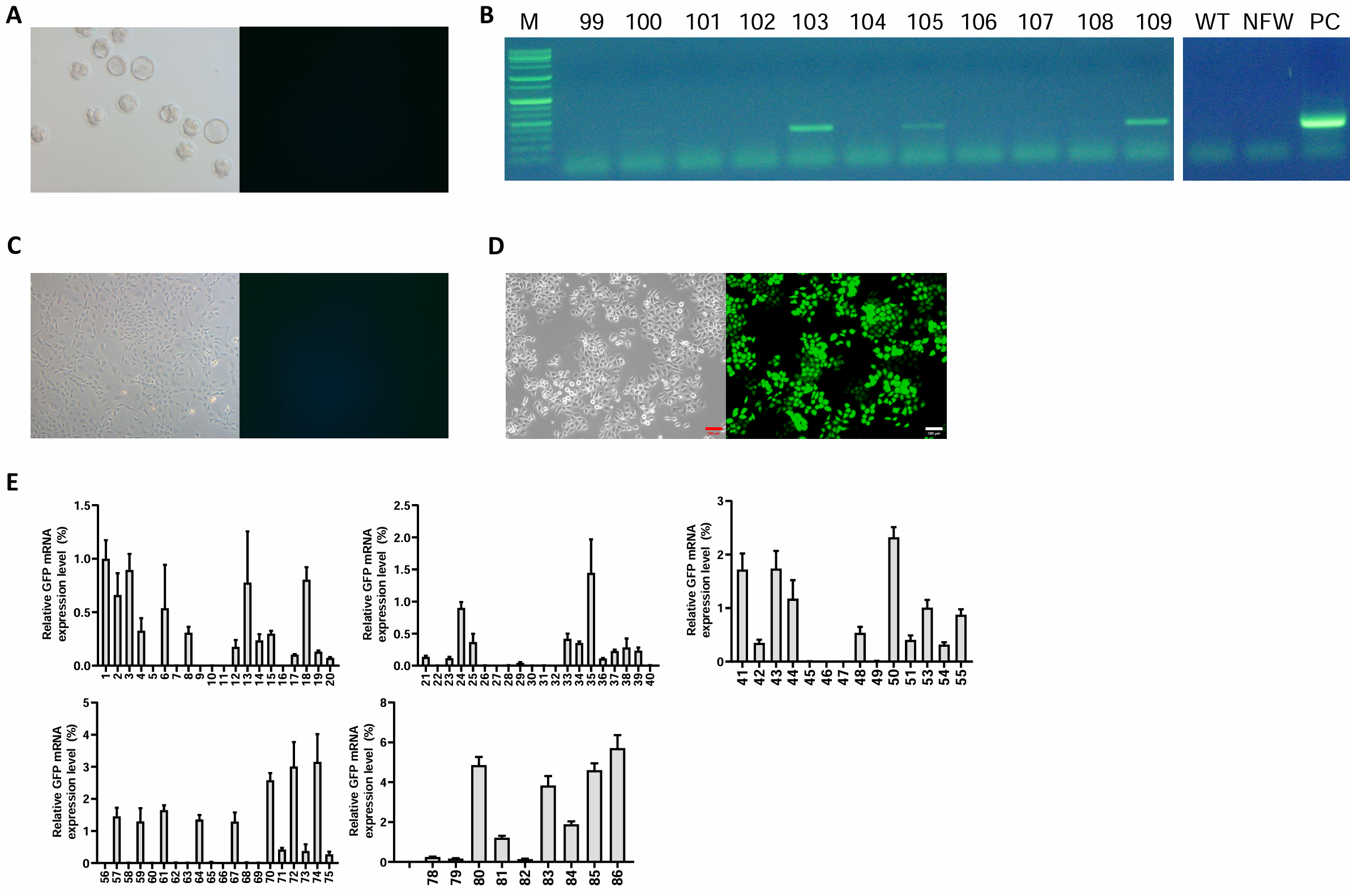


Supplementary Figure 1. PB-CAG-GFP silencing and PB-GFP rats RT-PCR. PB-CAG-GFP silencing and PB-GFP rats RT-PCR. (A) Representative images of PB-CAG-GFP embryo. (B) PCR image of PB-CAG-GFP rats. Some of offspring (#103, 105, 109) were PCR positive. Two separate parts of one image are cropped and grouped. Full size images are available in accompanying supplementary information. WT, wild-type rat, NFW, nuclease free water, negative control, PC, positive control of PCR. (C) Picture of primary fibroblast of PB-CAG-GFP rat (#109). (D) Picture of Hela cells transfected with PB-CAG-GFP and Ef1α-TASE vector, scale bars = 100µm. (E) Full RT-PCR results of PB-EF1α-GFP rats.

Supplementary figure 2


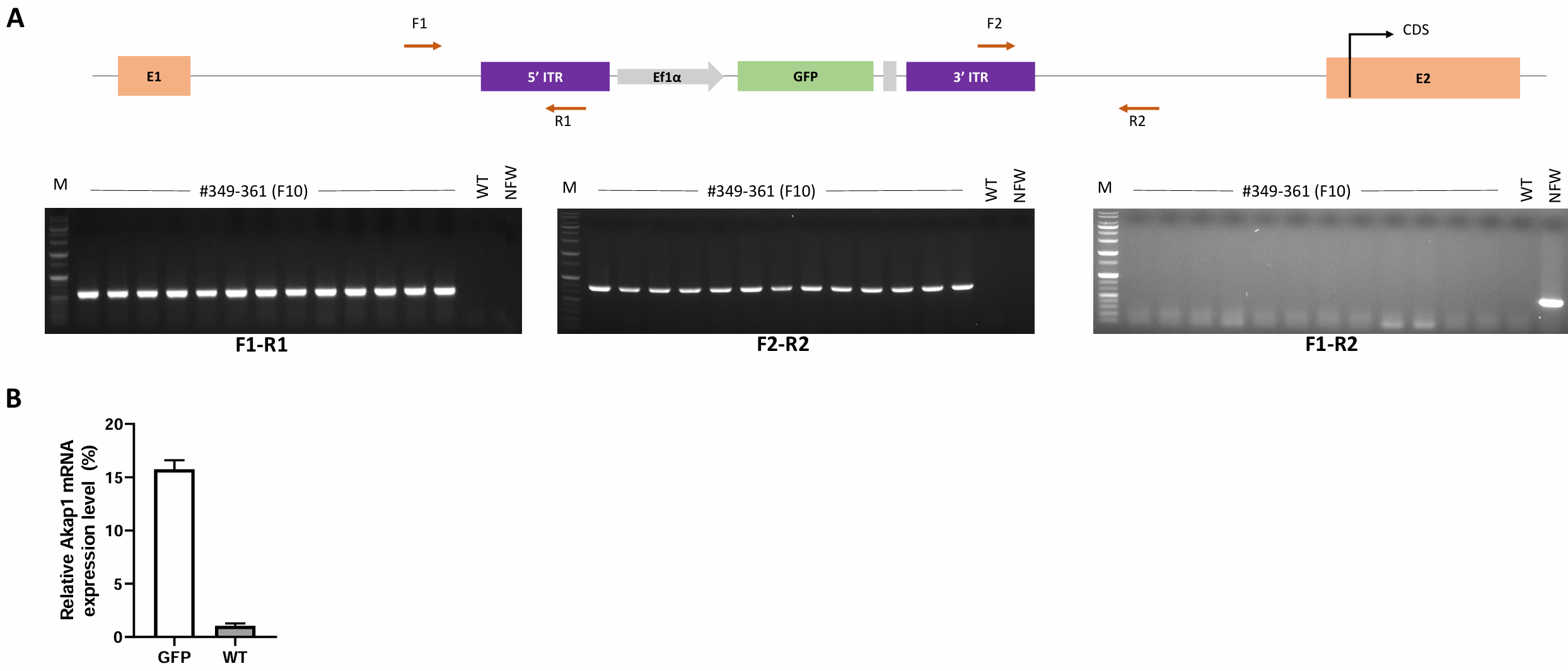


Supplementary Figure 2. Validation of long-term germline transmission of PB-EF1α-GFP in Akap1 locus. (A) Top, graphical image of PB-EF1α-GFP integration site in Akap1 gene and primer location. Bottom left, PCR image of F10 GFP rats using F1-R1 primers. Bottom middle, PCR image of F10 GFP rats using F2-R2 primers. Bottom right, PCR image of F10 GFP rats using F1-R2 primers. E1 of Akap1 gene: Exon 1, F1: Forward primer #1, R1: Reverse primer #2, F2: Forward primer #2, R2: Reverse primer #2, E2: Exon2 of Akap1 gene. (B) qRT-PCR result of Akap1 gene expression in GFP and wild type rat.
